# Detection and Clinical Significance of Intratumoral *EGFR* Mutational Heterogeneity in Chinese Patients with Advanced Non-Small Cell Lung Cancer

**DOI:** 10.1371/journal.pone.0054170

**Published:** 2013-02-13

**Authors:** Hua Bai, Zhijie Wang, Yuyan Wang, Minglei Zhuo, Qinghua Zhou, Jianchun Duan, Lu Yang, Meina Wu, Tongtong An, Jun Zhao, Jie Wang

**Affiliations:** 1 Key Laboratory of Carcinogenesis and Translational Research (Ministry of Education), Department of Thoracic Medical Oncology, Peking University Cancer Hospital & Institute, Beijing, China; 2 Department of Thoracic Surgery, Tianjin Medical University General Hospital, Tianjin, China; Queen Elizabeth Hospital, Hong Kong

## Abstract

**Purpose:**

This study evaluated occurrence and potential clinical significance of intratumoral *EGFR* mutational heterogeneity in Chinese patients with non-small cell lung cancer (NSCLC).

**Materials and Methods:**

Eighty-five stage IIIa-IV NSCLC patients who had undergone palliative surgical resection were included in this study. Of these, 45 patients carried *EGFR* mutations (group-M) and 40 patients were wild-type (group-W). Each tumor sample was microdissected to yield 28–34 tumor foci and Intratumoral *EGFR* mutation were determined using Denaturing High Performance Liquid Chromatography (DHPLC) and Amplification Refractory Mutation System (ARMS). *EGFR* copy numbers were measured using fluorescence in situ hybridization (FISH).

**Results:**

Microdissection yielded 1,431 tumor foci from *EGFR* mutant patients (group-M) and 1,238 foci from wild-type patients (group-W). The *EGFR* mutant frequencies in group-M were 80.6% (1,154/1,431) and 87.1% (1,247/1,431) using DHPLC and ARMS, respectively. A combination of *EGFR*-mutated and wild-type cells was detected in 32.9% (28/85) of samples by DHPLC and 28.2% (24/85) by ARMS, supporting the occurrence of intratumoral heterogeneity. Thirty-one patients (36.5%) were identified as *EGFR* FISH-positive. Patients harboring intratumoral mutational heterogeneity possessed lower *EGFR* copy numbers than those tumors contained mutant cells alone (16.7% vs. 71.0%, *P*<0.05). Among 26 patients who had received EGFR-TKIs, the mean *EGFR* mutation content was higher in patients showing partial response (86.1%) or stable disease (48.7%) compared with patients experiencing progressive disease (6.0%) (*P* = 0.001). There also showed relationship between progression-free survival (PFS) and different content of EGFR mutation groups (pure wild type EGFR, EGFR mutation with heterogeneity and pure mutated EGFR) (*P* = 0.001).

**Conclusion:**

Approximately 30% of patients presented intratumoral *EGFR* mutational heterogeneity, accompanying with relatively low EGFR copy number. *EGFR* mutant content was correlated with the response and prognosis of EGFR-TKIs.

## Introduction

Epithelial growth factor receptor-tyrosine kinase inhibitors (EGFR-TKIs) such as gefitinib and erlotinib had been applied broadly to the treatment of non-small cell lung cancer (NSCLC). Several reports have suggested that patients treated with EGFR-TKIs exhibit improved treatment efficacy and survival times when they carry activating mutations in *EGFR* [1]–[6]. However, Na et al reported that the female adenocarcinoma patients with EGFR sensitive mutation presented more postoperative recurrence and shorter survival than those with wild-type EGFR [7]. There also exist *EGFR*-mutated NSCLC patients who exhibit poor responses to EGFR-TKIs or who relapse following a period of disease control, suggesting that there are additional factors mediating the response to EGFR-TKIs. Secondary mutation (T790M) in exon 20 of *EGFR* as well as other genetic aberrances of EGFR related bypass and downstream pathways, such as, *C-MET* amplification [8]–[10], *IGF-1R* mutation [11] had been identified relative to TKIs drug resistance. However, about 30% of patients' resistance mechanisms remain unclear. Recently, intratumoral heterogeneity of *EGFR* mutations has garnered attention as a potential source of treatment failure and drug resistance to EGFR-TKIs [12], [13].

Tumorigenesis of lung cancer is a multistage process by which monoclonal cancer cells gradually become heterogeneous owing to clonal evolution and genetic/epigenetic instability. Although all malignant cells are thought to be derived from a common precursor cell, acquired genetic instability gives rise to subsequent generations expressing unique characteristics, such as activated oncogenes and tumor suppressor genes [14]. However, recent studies involving intratumoral genetic heterogeneity have generated contradicting results. Gerlinger et al (2012) [15] reported marked intratumoral heterogeneity with respect to somatic mutations in driver and passenger genes, which may foster tumor adaption and therapeutic failure via Darwinian selection. Snuderl et al (2011) [16] reported stable coexistence of heterogeneous clones possessing different receptor tyrosine kinase amplification (*EGFR, MET*, and *PDGFRA*) within the same tumor. As a driver gene, *EGFR* was suggested to be associated with resistance to EGFR-TKIs when mutations in this gene exhibited intratumoral heterogeneity. Our recent study (2012) [17] also indicated that *EGFR* mutation shift deriving from chemotherpy may be related to the heterogeneity of intratumoral *EGFR* mutation and to different chemosensitivity levels of mutant and wild-type cells, In contrast, Yatabe et al (2011) [18] reported that *EGFR* heterogeneity occurred extremely rarely in lung adenocarcinoma. These authors speculated that the heterogeneity observed in previous studies was an artifact resulting either from a mutant allele-specific imbalance and heterogeneously distributed *EGFR* amplification or from a difference in *EGFR* mutation detection sensitivity across different methods.

Based on the disparate results of the previously serial studies on intratumoral heterogeneity, we attempted to investigate *EGFR* mutation status by multi-focal microdissection analysis using different methods (DHPLC vs ARMS), explore the association of the intratumoral heterogeneity with *EGFR* copy number and imfluence of *EGFR* mutation contents on response of EGFR-TKI therapy for the patients with locally or advanced NSCLC.

## Materials and Methods

### Patients and specimens

All samples used in this study were obtained from a tissue bank at Department of Thoracic Medical Oncology, Peking University Cancer Hospital, which was established in June 1999 and have possessed around 1900 patients with tissues samples which had been genotyped for *EGFR* mutation status using routine methods (DHPLC). We selected patients from tissue bank in accordance with the following criteria: 1) histologically confirmed stage IIIa-IV NSCLC (pathology report); 2) had received palliative operational resection; 3) could provide sufficient primary tissue samples for microdissection and molecular analysis. The exclusive criteria included: 1) had tissues but was metastatic site samples; 2) the tumor cell content was too low to analysis. The palliative operational resections were defined as the operation performed in the patients with advanced NSCLC who had small intra-pulmonary nodules, solitary metastasis in single organ or pre-operative unidentified metastatic disease.

Finally, 85 patients met the above criteria and were included in this study which contained 45 samples typed as *EGFR* mutant (group-M) and 40 *EGFR* wild-type sample (group-W). All patients provided written informed consent for biomarker analysis. The study protocol was approved by the Institutional Ethics Committee at Peking University Cancer Hospital.

### Microdissection and DNA extraction

All specimens had been evaluated for *EGFR* mutation by DHPLC routinely and were sorted into 40 wild-type and 45 mutant-type samples. From each Formalin-Fixed Paraffin-Embedded (FFPE) block, one 15-μm-thick section was stained with hematoxylin and eosin (H&E).

To ensure the samples analyzed had more than 90% tumor contents, a protocol is routinely performed in our group: Firstly, tumor boundary on the section was drawn by two independent pathologists under microscopy and excluded the non-malignant tissues as soon as possible. Secondly, small foci (about 0.1 cm2 size) within tumor region were microdissected using Laser Microdissection System (LMC, Leica Wetzler, Germany) and assure every foci contain more than 90% tumor cells.

DNA was extracted from aliquots of microdissected samples using FFPE DNA Extraction Kit according to the manufacturer's instructions (OMEGA). DNA samples were examined for purity and concentration using a Nano Drop kit (Thermo Scientific) and were diluted to a working concentration of 10 ng/μl.

### EGFR mutation analysis by DHPLC and ARMS

The *EGFR* mutational statuses of genomic DNA samples derived from tumor microdissections were determined by applying both DHPLC and ARMS to each sample. DHPLC was performed as previously described [19], and ARMS was conducted using a DxS *EGFR* Mutation Test Kit, according to the manufacturer's recommendations (Amoy Diagnostics Co., LTD, China). Quantitative PCR (qPCR) reactions were performed using an Mx3000P Real-Time QPCR System (Stratagene, Agilent Technologies, USA).

### Semiquantitation of EGFR mutation heterogeneity

A semiquantitative DHPLC analysis of *EGFR* abundance was performed by calculating the ratio of peak heights between the mutant (M) and wild-type (W) products (ratio, M/W). The DHPLC analysis was limited to mutations in exon 19 because M and W peaks were separated completely but overlapped in exon 21 mutation detection.

### EGFR copy number detection

*EGFR* copy numbers were determined by fluorescence in situ hybridization (FISH) from bulk tissue using dual-color DNA probes (Beijing GP medical Tec., LTD, China). Tumor specimens were classified into six categories on the basis of FISH results according to the criteria of Cappuzzo [20]. Cytogenetic patterns were classified as FISH-negative if they displayed no or low genomic gain (*i.e*., ≤4 copies of *EGFR* in >40% of cells). Samples were classified as FISH-positive if they exhibited a high level of polysomy (≥4 copies of *EGFR* in ≥40% of cells) or if they displayed gene amplification, defined as the presence of tight *EGFR* gene clusters and a ratio of *EGFR*/chromosome 7 centromere of 2 or more per cell or 15 or more copies of *EGFR* per cell in at least 10% of analyzed cells.

### Statistics

χ2 test were used to analyze the association of mutation content with copy number. McNemar's test was applied to compare disparity of *EGFR* mutation heterogeneity between DHPLC and ARMS. Wilcoxon rank sum test was applied to compare the mutant abundance between the different mutation groups. Non-parametric tests was used to analyze the mutation content between different groups. Two-sided P values <0.05 were considered significant. Data evaluation was carried out with All calculations were performed using SAS Version 10.0 (SAS Institute, Inc., Cary, NC).

## Results

### Characterization of patients

During the period October 2005-December 2011, 85 patients were enrolled into this retrospective study. Histologic subtypes of each NSCLC sample were evaluated based on World Health Organization (WHO) criteria [21]. Adenocarcinoma was the most common histologic subtype (63 patients, 74.1%). Cancer staging was performed according to the UICC–AJCC-TNM system (version 7, 2009) [22]. Advanced disease (stages IIIb – IV) was identified in 61.2% of enrolled patients. Patient data are summarized in Table 1. Forty-five patients were confirmed to be *EGFR* mutant type (group-M), including 25 patients with exon 19 deletions (group-M/E19) and 20 patients with exon 21 mutations (group-M/E21).

**Table 1:** Patient characteristics.

Characteristic	Number
**Age (yr)**	
** <60**	40
** ≥60**	45
**Gender**	
Male	47
Female	38
**Histology**	
Adenocarcinoma	63
Squ carcinoma	10
Ade-Squ carcinoma	5
Other	7
**Stage**	
IIIA	33
IIIB	17
IV	35
**Smoking**	
Yes	39
No	46
**Mutation status**	
Mutation	45
wild	40

### Heterogeneity of EGFR mutation detected by DHPLC and ARMS

Tumor microdissection yielded 2,699 foci, including 1,238 foci from wild-type *EGFR* (group-W) and 1,431 group-M cases. The overall mutant frequency in group-M was 80.6% by DHPLC (1,154/1,431) and 87.1% (1,247/1,431) by ARMS. Mutant frequencies varied widely throughout individual tumors, ranging between 5%–100% by DHPLC and 1%–100% by ARMS. Group-M samples were subdivided by mutation content as follows: 1) pure *EGFR* mutation (100%) or no mutational heterogeneity was detected in 21 cases by DHPLC (12 group-M/E19, 9 group-M/E21) and in 31 cases by ARMS (20 group-M/E19, 11 group-M/E21); 2) moderate mutant content (≥50%) or moderate-level heterogeneity was detected in 16 cases by DHPLC (10 group-M/E19, 6 group-M/E21) and in 9 cases by ARMS (2 group-M/E19, 7 group-M/E21); and 3) low mutant content or high-level heterogeneity (<50%) were detected in 8 cases by DHPLC (3 group-M/E19 and 5 group-M/E21) and in 5 cases by ARMS (3 group-M/E19 and 2 group-M/E21).

**Figure 1:**
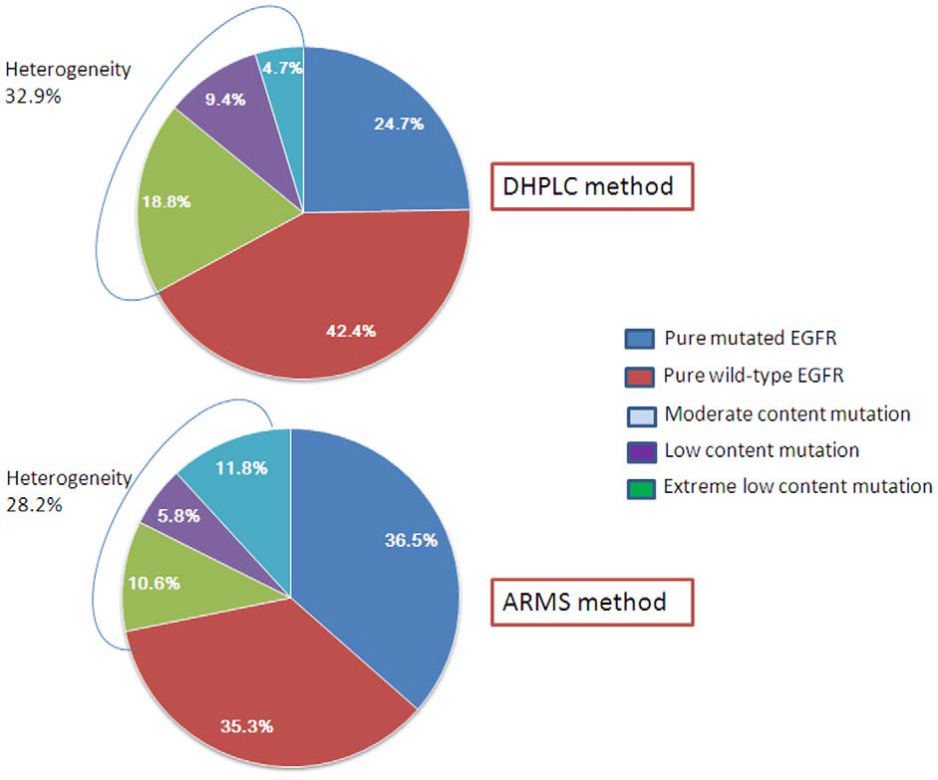
Heterogeneity of EGFR mutation by DHPLC and ARMS. The different color of pie represent different EGFR mutation heterogeneity. The left three parts (lightblue, purple and green) of each pie chart represent percentage of cases with EGFR heterogeneity.

Among the 40 group-W cases, 4 cases displayed low mutant frequencies ranging from 5.0% to 8.0% when microdissected tumor foci were analyzed by DHPLC. Three of these cases carried an *EGFR* exon 19 mutation, and one case carried an *EGFR* exon 21 mutation. ARMS confirmed these 4 cases and identified 6 additional cases showing very low mutant frequencies ranging from 1.0% to 5.0%. By combining the group-M cases carrying both wild- and mutant-type *EGFR* cells and the group-W cases containing mutant cells at low frequency, 32.9% (28/85) and 28.2% (24/85) of samples were identified to carry intratumoral *EGFR* mutational heterogeneity by DHPLC and ARMS, respectively. Difference of intratumoral *EGFR* mutational heterogeneity identified by two methods was not statistical significance (*P* = 0.031, McNemar's test) (Figure 1).

### Semiquantitative analysis of exon 19 mutation by DHPLC

**Figure 2:**
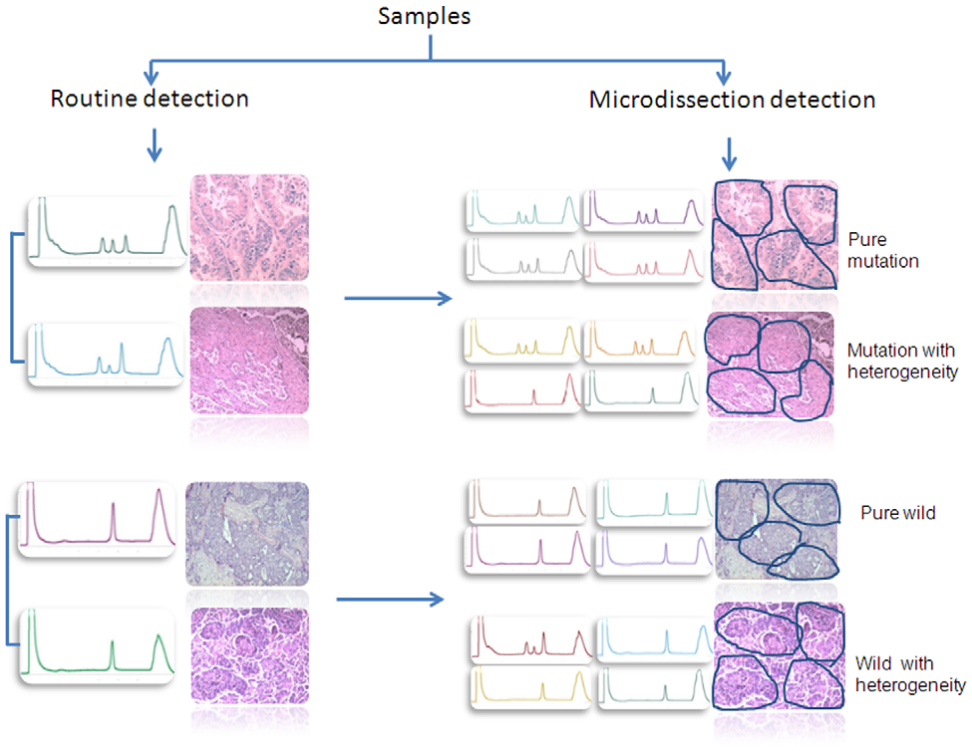
A representative example of intratumoral *EGFR* genetic heterogeneity. The left graph showed routine EGFR mutation status detection by DHPLC and corresponding bulk tissue. The right graph represent EGFR mutation heterogeneity by microdissection. The above two panels represent mutant EGFR in bulk tissues showed pure mutation and mutation with heterogeneity by microdissection, respectively. The below two panels represent wild EGFR in bulk tissues with pure wild-type EGFR or mixed with low frequency of mutation cells by microdissection.

We also measured semiquantitatively the *EGFR* mutant abundance by calculating the M/W peak height ratio from the DHPLC graph. We limited this analysis to the exon 19 deletion because the corresponding M and W peaks do not overlap under undenatured conditions (50°C) (Figure 2). The median M/W ratios of exon 19 were 2.43 (range, 0.40–18.15) among group-M samples and 0.12 (range, 0.06–0.22) among group-W samples (*P* = 0.005). Under partly denatured conditions (62.2°C), the M and W peaks corresponding to the exon 21 substitution overlapped, precluding any determination of their relative heights.

### EGFR copy number

**Figure 3:**
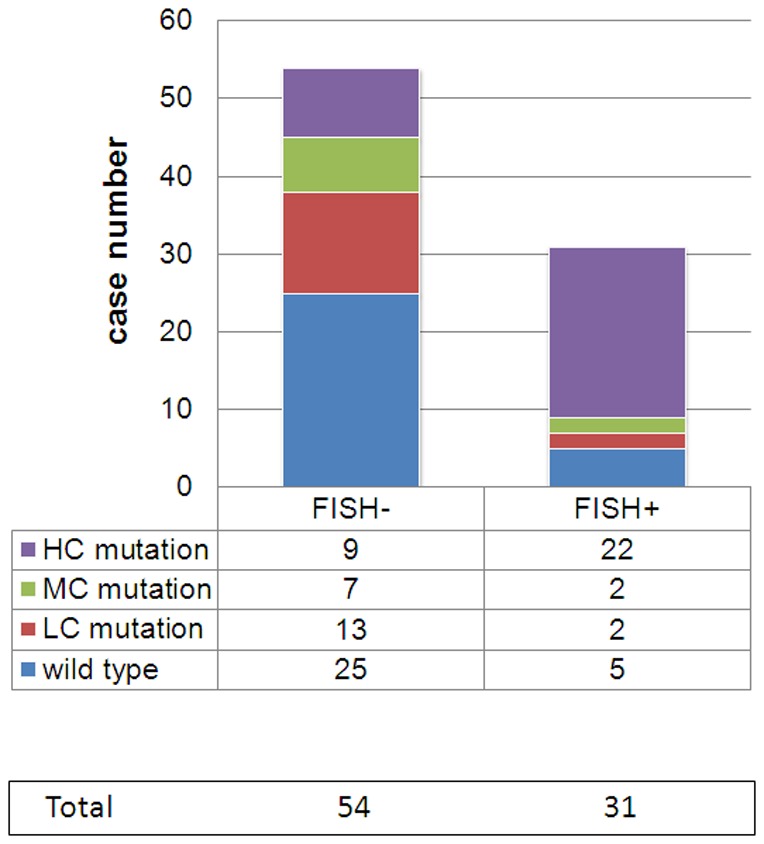
Extent of *EGFR* heterogeneity as measured by FISH. The various *EGFR* mutation contents are depicted in the legend.

**Figure 4:**
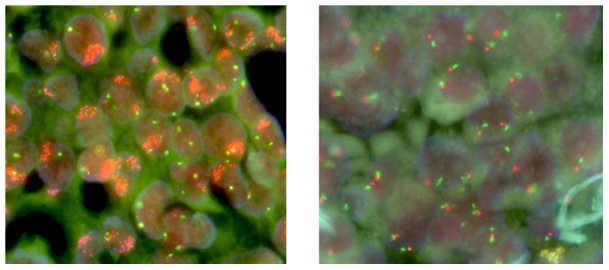
*EGFR* copy number was determined by FISH. Evaluation of EGFR gene copy number by FISH was done using the *EGFR* (orange)/CEP 7 (green) probe (Beijing GP medical Tec., LTD, China). Panels illustrate tumor specimens representing gene amplification (A) and disomy (B).

FISH analysis was conducted on 85 tumor samples to measure *EGFR* copy numbers. Thirty-one cases (36.5%) were considered FISH-positive. The association of *EGFR* mutational heterogeneity as detected by ARMS with *EGFR* copy number is described below. Among the 31 patients with 100% *EGFR*-mutated cells (no heterogeneity), 71.0% (22/31) were classified as FISH-positive (high polysomy or gene amplification). By contrast, the low *EGFR*-mutated group exhibited approximately 13.3% FISH-positivity (2/15, including 10 low-frequency mutant cases in group-W and 5 low-frequency mutant cases in group-M), and the moderate *EGFR*-mutated group displayed approximately 22.2% (2/9) FISH positivity (*P*<0.05; Figure 3 and 4) which were similar to that detected in 30 patients with wild-type *EGFR* from whom microdissection foci were analyzed using ARMS (5/30, 16.7%, *P* = 0.75).

### Heterogeneity by histological type and stage

Adenocarcinoma was the most common histological pattern identified in this study (74.1% of subjects); this was expected in a predominantly surgical series. The positivity of *EGFR* mutation in adenocarcinoma, as confirmed by microdissection analysis, was 91.8%. The M/W ratio was 2.57 (range, 0.13–18.15). In comparison, the *EGFR* mutation frequency was lower among other histological patterns (70.9%), and only 3 cases carried the *EGFR*19 mutation with M/W ratio 1.53. Based on the UICC-AJCC-TNM tumor staging system, 50 tumors were classified as stage IIIa and IIIb (locally advanced) and 35 were classified as stage IV (advanced). No significant differences in the mutation positive rate and abundances were observed between locally advanced and advanced stages NSCLC (rate, 87.7% vs. 86.3%, *P* = 0.875; M/W ratio, 2.12 vs. 2.86, *P* = 0.662).

### Impact of EGFR mutational heterogeneity on response to EGFR-TKIs

Among 85 cases, 26 patients received EGFR-TKIs as first-line or multi-line therapies. 9 patients exhibited a partial response (PR), 7 had stable disease (SD), and 10 experienced progressive disease (PD). According to ARMS results, the mean mutant content was 86.1% (247/287) in the PR group, 48.7% (110/226) in the SD group, and 6.0% (19/317) in the PD group, and with a significant difference (*P* = 0.001).

We divided 26 patients into three subgroups according to EGFR mutation heterogeneous status, which were “pure wild type EGFR”, “EGFR mutation with heterogeneity” and “pure mutated EGFR”. The PFS were 3.01 months (95% CI 0.51–5.52), 11.35 months (95%CI 6.37–15.21) and 16.21 months (95%CI 8.21–25.19) for the three groups, respectively (P = 0.001).

## Discussion

Intratumoral *EGFR* mutation homogeneity has long been assumed in lung cancers. As such, the determination of a patient's *EGFR* mutation status was interpreted using qualitative methods. However, only a fraction of patients harboring *EGFR* mutations respond to EGFR-TKIs, suggesting that additional factors beyond EGFR mutation contribute to a patient's drug response. In addition to several related biomarkers (*e.g*., K-ras, T790M, C-met), intratumoral *EGFR* mutational heterogeneity has been proposed as a candidate mediator of the resistance to EGFR-TKI therapy, although the existence of such heterogeneity has been disputed [15]–[17]. In the present study, we observed significant intratumoral mutational heterogeneity using both DHPLC and ARMS methods.

One of the controversies on intratumoral heterogeneity of *EGFR* mutation is whether the heterogeneity attributes to using a low sensitive detection method, especially when mutated signal is below the threshold of detection. To exclude the possibility, we utilized two testing methods, DHPLC and ARMS, to evaluate the *EGFR* mutation status in the microdissected intratumoral foci. ARMS is thought as a high sensitive assay for *EGFR* mutation detection. Whereas DHPLC method has not been widely used for *EGFR* mutation analysis, however, it's high sensitivity and specificity have been shown in our and other studies (detection limit 3%∼10%) [19], [23]–[26]. The results showed the part of specimens from locally advanced and advanced NSCLC presented the coexistence of *EGFR* mutant and wild-type cells regardless of the DHPLC or ARMS being used, suggesting that the intratumoral *EGFR* mutated heterogeneity indeed existed and didn't derive from the bias of detection methods or low intratumoral mutated frequencies.

In order to better characterize intratumoral heterogeneity of *EGFR*, we microdissected bulk tumor tissue to obtain 28–34 foci per tumor and analyzed each focus for *EGFR* mutation status using qualitative and semi-quantitative methods. Most of the microdissection foci were identified as *EGFR* mutant-type with *EGFR* mutation contents ranging from 1% to 100%. Theoretical and experimental studies have reported that cancer cells of the same genotype locate contiguously [27]. Analysis of a small sample excised from tumor tissue would likely indicate a genetically identical population of cancer cells. However, our analysis of *EGFR* mutation statuses from numerous intratumoral foci indicated heterogeneity. We excluded non-malignant sites by microscopic visualization, and every microsampled specimen was cross-checked to confirm that the percentage of tumor tissue was at least 90%, ensuring that microdissected areas were not contaminated by non-cancerous cells. Thus, our data confirmed the existence of intratumoral *EGFR* mutational heterogeneity.

Yatabe et al. (2011) [17] reported that heterogeneous distributions of *EGFR* mutations in lung adenocarcinoma were extremely rare. The authors suggested that the tumor heterogeneity reported by others was actually a pseudoheterogeneity resulting from a mutant allele-specific imbalance (MASI) or from heterogeneously distributed *EGFR* amplification. Several studies supported the occurrence of MASI in EGFR-mutated tumor cells, and this phenomenon is associated with increased mutant allele transcriptional activity [28]–[30]. *EGFR* mutations, gene copy number gains, and MASI occurring together in tumor cells appear to synergize to effect a more malignant phenotype than these alterations individually [28]–[30]. We observed an elevated *EGFR* copy number among patients with pure *EGFR* mutations, suggesting that a high frequency of *EGFR* mutations occur frequently with an elevated *EGFR* copy number (MASI). However, in patients displaying intratumoral heterogeneity, *EGFR* copy number was lower than in patients with pure *EGFR* mutant tumors. The possible explain is that heterogeneous tumors contained not only *EGFR* mutant cells but also wild-type cells, and selective amplification of mutant alleles could be diluted by wild-type alleles, giving rise to a relatively low *EGFR* copy number.

Among bulk tumors harboring wild-type *EGFR*, 10 of 40 cases exhibited far lower mutant frequencies (5–8%) via microdissection analysis compared with the 45 patients harboring mutant *EGFR*. This so-called “false negativity” measured from bulk tissue might be owing to the inability of DHPLC to detect trace amounts of mutant DNA when few mutant cells are contained in a specimen. We also semiquantitatively determined mutant abundance by calculating the ratio of peak heights (M/W) from the DHPLC graph. According to our data, the median M/W ratio ranged from 0.13 to 18.15 in *EGFR* mutant cases and from 0.06 to 0.22 in wild-type cases, which suggests that cancer cells in bulk tumor tissue usually contain mutant and non-mutant types simultaneously. To our knowledge, this is the first study applying the M/W ratio to semi-quantitatively determine *EGFR* mutation status and infer intratumoral heterogeneity.

Only 26 patients received EGFR-TKIs, which limited the statistical power of this arm of the study. However, we detected a significantly higher mutation rate in foci from PR and SD patients compared with PD patients. These results were consistent with several other studies. Taniguchi et al [31] analyzed the relationship between *EGFR* heterogeneity and the response to EGFR-TKIs by microdissection in early-stage NSCLC tumors, and reported that patients with a greater *EGFR* mutation frequency were more sensitive to EGFR-TKIs and showed longer progression-free survival times than patients with low *EGFR* mutation rates. We speculated that rapid tumor progression may occur in samples with a low percentage of *EGFR* mutant cells, owing to the fact that the apoptotic *EGFR* mutant cells responding to EGFR-TKI were outnumbered by proliferating non-mutant cells. Based on this speculation, we suggest that intratumoral heterogeneity may be an important factor contributing to EGFR-TKI resistance. Patient responses may by improved by administering a combination of EGFR-TKI therapy and other therapeutics capable of clearing non-*EGFR* mutant tumor cells in a simultaneous or sequential manner.

Overall, our findings suggest that patients with advanced lung cancer harbored marked EGFR mutational heterogeneity. The most important clinical significance of EGFR mutational heterogeneity may be able to explain resistance mechanism of EGFR-TKI. Secondary, intratumoral heterogeneity of EGFR mutation also indicate that single point or single time biopsy might not be optimal method to determine personalized EGFR-TKI therapy. Combinatorial histologic and genetic approaches using information on survival and response profiles may provide better inferences for effective disease prevention and cure in the future.
